# The Influence of COVID-19 Pandemic on Physical Health–Psychological Health, Physical Activity, and Overall Well-Being: The Mediating Role of Emotional Regulation

**DOI:** 10.3389/fpsyg.2021.667461

**Published:** 2021-08-16

**Authors:** Jianhui Dai, Xuehui Sang, Rashid Menhas, Xia Xu, Sumaira Khurshid, Sajid Mahmood, Yu Weng, Jiaai Huang, Yuwei Cai, Babar Shahzad, Waseem Iqbal, Maryam Gul, Zulkaif Ahmed Saqib, Muhammad Nurul Alam

**Affiliations:** ^1^School of Physical Education and Sports, Soochow University, Suzhou, China; ^2^Research Center of Sports Social Sciences, School of Physical Education and Sports, Soochow University, Suzhou, China; ^3^Hubei Key Laboratory of Sport Training and Monitoring, Wuhan Sports University, Wuhan, China; ^4^School of Education and Science, Neijang Normal University, Neijiang, China; ^5^Department of Zoology, Hazara University, Mansehra, Pakistan; ^6^Graduate School, Wuhan Sports University, Wuhan, China; ^7^School of Energy, Soochow University, Suzhou, China; ^8^Department of Applied Psychology, Lahore College for Women University, Lahore, Pakistan; ^9^College of Management, Shenzhen University, Shenzhen, China; ^10^Academy for Research Skills Development (AFRSD), Dhaka, Bangladesh

**Keywords:** COVID-19, lockdown, HRQoL, emotional regulation, physical health, psychological health, physical activity, overall well-being

## Abstract

**Background:** Highly infectious respiratory disease COVID-19 emerged in Wuhan, China, and spread worldwide. Different measures have been adopted worldwide to contain the COVID-19, and these measures have various impacts on health-related quality of life (HRQoL). This study aimed to assess the impact of the COVID-19 pandemic (CP) and lockdown policy on physical health (PH)–psychological health (PsH), physical activity (PA), and overall well-being (OW) in the context of HRQoL, exploring the mediating role of emotional regulation (ER).

**Method:** The current study was conducted in two provincial cities of China. An online survey was conducted in both the cities to collect the data. After quantifying the data, a total of 2,200 respondents data were analyzed through appropriate statistical techniques.

**Results:** The study results indicate that CP was found significantly and negatively related to PH (β = −0.157, *t* = 9.444, *p* < 0.001). A significant relationship was found between CP and PsH (β = 0.779, *t* = 45.013, *p* < 0.001). The third prediction revealed a significant negative relationship between the CP and OW (β = −0.080, *t* = 5.261, *p* < 0.001). The CP and PA had a significant negative relationship (β = −0.047, *t* = 3.351, *p* < 0.001).

**Conclusion:** The PH, PsH, and OW of the Chinese people were affected due to the CP and lockdown measures. It is suggested that ER intervention reduces the negative psychological impacts for improving quality of life. ER can function one's sentiments in their social environment effectively for quality of life.

## Introduction

In early December 2019, in Wuhan, Hubei Province, China, a highly infectious respiratory disease, that is, Coronavirus (COVID-19) emerged and extended globally. On March 11, 2020, this newly emerged viral infection was declared a worldwide health emergency by World Health Organization (WHO) (Rogowska et al., 2020). According to the WHO, the COVID-19 has spread worldwide, and 213 countries are taking multiple measures to contain the COVID-19 by their governments. On January 23, 2020, the governments implemented various measures, such as the lockout of entire cities, travel warning regulation, and home medical observations to prevent and control the viral transmission (Anna, 2020). Because of the threats of COVID-19 pandemic (CP) to health care systems and society at large, and to reduce the incidence of novel infections and flatten the COVID-19 infection curve, a global mass home-confinement directive has been implemented in many countries, most of which entail social isolation and quarantine. Social isolation and quarantine can be the main stressors that can lead to emotional distress and other unpredicted mental health and psychological consequences (Hossain et al., 2020). Pandemics have different stages and come in waves with various severe impacts on human health and society. CP is also coming in waves in which the virus pathogen is becoming more dangerous, creating pressure on public health facilities worldwide. Many countries worldwide introduced different policies that include total lockdown, smart lockdown, health monitor system, and quarantine to contain the virus (Alwan et al., 2020). Public health experts review the CP-related lockdown policies (LPs) daily to ensure public safety (Al Zobbi et al., 2020; Plümper and Neumayer, 2020).

Studies have reported loneliness, anxiety, boredom, anger, denial, depression, insomnia, harmful substance use, despair, self-harm, and suicides in quarantined individuals (Li et al., 2020; Wang et al., 2020). Furthermore, COVID-19 physical symptoms (such as cough, hypoxia, and fever) along with side effects of recommended medicines (corticosteroids) may lead to more psychological distress and anxiety (Wang et al., 2020). Researchers reported that various psychiatric disorders could be found in individuals, for instance, anxiety disorders, self-blame, guilt, post-traumatic stress disorder, depressive disorders, delirium, somatic symptoms, panic disorder, psychosis, and even suicide (Goyal et al., 2020). Furthermore, our findings are similar in the context of emotional regulation (ER) with the studies of Li et al. (2021) and Cheng et al. (2006), which were carried out among the Chinese population and concluded that socio-behavioral restrictions are negatively associated with the health-related quality of life (HRQoL). There is also a negative impact on emotions on time spent under quarantine measures. It is consistent with previously published work on the mental health effects of the lockout of COVID-19 (Ozamiz-Etxebarria et al., 2020). Several studies reported adverse impacts, such as depression, loneliness, anxiety, and post-traumatic stress due to the CP. The suddenly declared pandemic has drastic negative effects on every segment of human society in the socio-psychological and physical paradigm. Muzi et al. (2021) conducted a study on Italian adolescents and reported that the teenagers might have used social media disorder symptoms to express CP adverse effects. Teenagers during pandemic showed lower internalizing but higher other issues (such as excessive drinking and self-destructive behaviors) and more problematic social media usage than pre-pandemic samples.

### COVID-19 Lockdown—Health and Well-Being

HRQoL, participation in physical activity (PA), and perceived mental stress among Chinese adults are significantly related to the CP. Physical inactivity and sedentary sitting time have been increased during home confinement among Chinese people. Long-term sitting has also proved to negatively impact well-being and quality of life (Qi et al., 2020). Lockdown policy (LP) implementation across China has generated many socio-psychological problems for the Chinese people in every segment of their lives. During the period of lockdown, people were confined to their homes to contain the pathogen. Domestic confinement has a long-lasting psychological and well-being effect. Chinese people were confronted by anger, boredom, and loneliness during home confinement, and psychological problems, such as depression, stress, and anxiety increased (Duan and Zhu, 2020). Mental health and quality of life among Chinese adults have been impacted negatively by the CP (Zhang and Ma, 2020). Home isolation has adverse socio-psychological effects on physical and mental health. Long-term isolation causes negative feelings, cognitive decline, and discomfort (Hawkley and Capitanio, 2015). The daily routine and lifestyle of Chinese citizens would inevitably be interrupted by restrictions on travel and outdoor leisure. Individuals were also less physically involved, more sedentary, and more depressed, which may pose severe protection and well-being risks (Chen et al., 2020). HRQoL has been affected due to the socio-psychological impacts of COVID-19 and caused a severe threat to global public health (Tsamakis et al., 2020). People with personality disorders may be particularly vulnerable to negative psychological impacts of the CP. ER skills appear to be a potential target for therapies targeted at reducing negative consequences (Velotti et al., 2021a,b).

### COVID-19—Emotional Regulation for Health and Well-Being

Emotions refer to an event-focused affective state, an intricate pattern of the reaction involving elements of experience, actions, and physiology (Sander et al., 2013). The advent of the CP can exacerbate these two psychological aspects and make people feel endangered. People interpret risk cognitively and respond to it emotionally. Risk beliefs are often the source of negative emotions and psychological distress (Leppin and Aro, 2009). The social and emotional reaction to the epidemic of COVID-19 is multidimensional. Furthermore, it depends not just on external factors but also on personal and innate components (Brooks et al., 2020). Emotions and feelings play a vital role in response to the sudden phenomena and reshape our understanding about how to cope with the negative impacts of different situations on our socio-psychological and health-related QoL. Emotion regulation is helpful in the maintenance of health behavior during the CP. Positive health behavior through ER is a catalyst for PA and psychological well-being (Julie, 2020). In empirical models of well-being, human growth, interpersonal processes, psychopathology, and decision making, emotions play a pivotal role (Ekman and Davidson, 1994; Saarni, 2008). Previous pandemics, such as Ebola, H1N1, and SARS evidence show that public emotional response is associated with risk perception (Yang, 2016). During the early stages of the COVID-19 outbreak in China, a nationwide survey found that ~27.9% of the participants had depression symptoms, and 31.6% had anxiety symptoms, which further leads to emotional sensitivity (Shi et al., 2020). ER is an effective way to maintain healthy behavior in particular circumstances. ER processes have long been known as a trans-diagnostic factor in various psychological problems (Sloan et al., 2017). ER theories indicate that the ability to control one's own emotions is necessary for psycho-social health (Gross and Munoz, 1995). Emotional stability requires explicit or implicit attempts to control the emotional perception, expression, length, and magnitude (Gross, 1998). A significant factor in raising or decreasing the risk of poor physical and PsH should be controlling feelings when people face the challenges of the pandemic (Low et al., 2020). PA is a catalyst for healthy living and plays a pivotal role in preventing different diseases (Saqib et al., 2020).

### Statement of the Study

**Figure 1:**
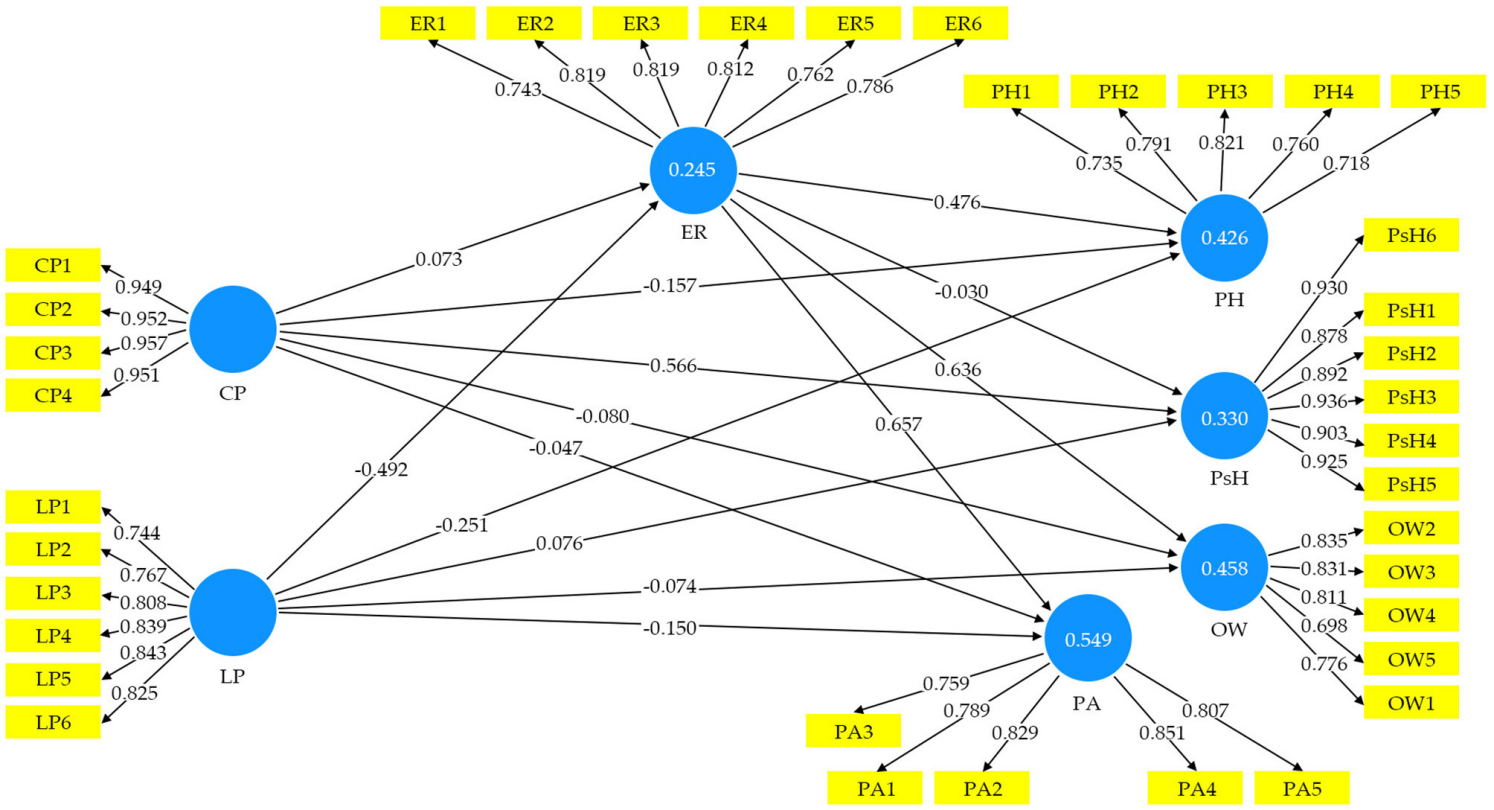
Conceptual model.

Significant adverse impacts have been observed due to the CP, which undermines the overall health and well-being. Preventive measures, such as home isolation during mandatory lockdown to contain the pathogen or the virus are used effectively to manage socio-economic life safely (Holmes et al., 2020). Regulation of emotion is significant in maintaining psychological and physical health (PH) during home isolation and lockdown during the pandemic period. People use emotions to add sense to their interactions and organize themselves and the people they communicate with (Duy and Yildiz, 2014). ER is also a vital principle that allows people to manage their emotional states and maintain healthy relationships with their environment (Gross, 2013). The CP created a fearful and panicked environment that has negative socio-psychological impacts on human lives. Management and regulation of emotions are the best ways to keep positive attitude and behavior toward a healthy quality of life. The pandemic created a crisis across the globe, and emotions are vital to human lives, which give direction to maintain socio-psychological and PH well-being. This study aimed to assess the impact of the CP and LP on PH–psychological health (PsH), PA, and overall well-being (OW) in the context of HRQoL, exploring the mediating role of ER. HRQoL through a mediating role of ER during CP explored PH, PsH, PA, and OW. Every person is experiencing COVID-19 in various ways and has different emotional reactions to manage OW. Regulation of emotions applies to people controlling their emotional perceptions and altering emotional expressions in the face of extreme or negative feelings, intentional behavioral improvement, and regulation of affective states (Leahy et al., 2011). It is especially true for those who have low self-control levels in maintaining their emotions and have drawn attention to reliance on others to manage their negative emotions. For persons who tend to emotional control issues feeling lonely may be extremely motivating. Humans may be able to express themselves based on the information they are processing through emotions. The grand theory of Aristotle proposed that emotions were sentiments associated with happiness or suffering, such as wrath, joy, fear, love, hatred, desire, and confidence (Bound, 2018). ER to negate the negative emotions and prevent psychological issues is the best way among the masses during the CP (Velotti et al., 2021a,b). The relationship between different variables of the study has been shown in Figure 1. The ER strategies have significant impacts on PH and PsH. ER and physical health well-being have an essential association under particular circumstances. The CP is posing a unique circumstance globally, and people are facing numerous kinds of challenges in every segment of life. In the paradigm of COVID-19, examining the relationship between ER, PH, PsH, PA, and OW have vital implications for health and routine life functioning.

## Materials and Methods

### Study Locale

**Figure 2:**
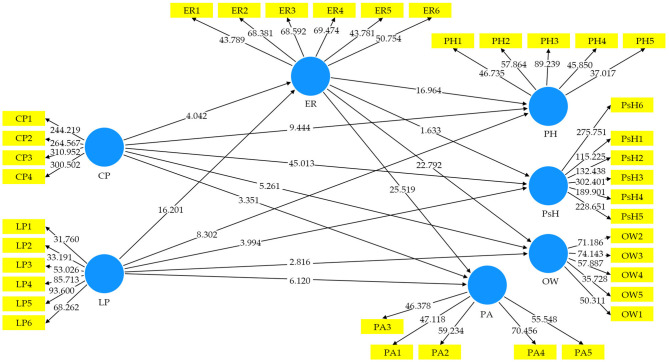
Study area.

The present study was conducted in two cities of China; Wuhan, Hubei Province, and Suzhou, Jiangsu Province, as shown in Figure 2. The study upheld the standards of the World Medical Helsinki Policy. Therefore, the Ethical Committee of Soochow University, Suzhou, Jiangsu and Wuhan Sports University, Wuhan, Hubei, approved the study.

### Participants

The current study population was Wuhan, Hubei Province, and Suzhou, Jiangsu Province city residents (+18) in the cities during the lockdown period. A convenience sampling method was used to collect the data by conducting an online survey in both the cities. A total of 2,280 respondents replied to the online survey questionnaire. After quantifying the data, a total of 2,200 respondents from Suzhou (1,034) and Wuhan (1,166) were included for the final data analysis. The answers of the 80 respondents were excluded because of incomplete information. According to the table statistics, most of the survey participants (~53.0%) lived in the Wuhan city, Hubei Province, where the first lockdown was implemented due to the severity of the CP and ~47.0% lived in Suzhou city, Jiangsu Province, during the lockdown period.

### Instrument and Data Collection

The impacts of CP and LPs were assessed in the context of HRQoL in association with ER, PH, PsH, PA, and OW. An online questionnaire survey method was used to collect the primary data from the targeted population in both Wuhan and Suzhou cities. The survey method was used to collect the primary data in the current study. The questionnaire was developed after reviewing the variables related to previous research studies regarding SARS and influenza outbreaks (Rubin et al., 2010). The questionnaire was pre-tested in both cities (Wuhan and Suzhou) targeted population before conducting the final survey for data collection. After pre-testing the questionnaire, some questions were amended and improved the wording of the comprehensive questionnaire for getting the best response rate from the study participants. An online survey was conducted in both Wuhan, Hubei Province, and Suzhou, Jiangsu Province cities from July 9 to August 10, 2020, to evaluate the CP and LPs and the HRQoL. The questionnaire was based on closed-ended 5-point Likert scale questions regarding the respondents' demographic information, CP, COVID-19 LP, ER, PH, PsH, participation in PA, and OW during the COVID-19 lockdown period. An informed consent received from all the study participants after informing about the purpose of the study. The researchers performed the quality check (accuracy, relevancy, and completeness) of the data collected anonymously. It was guaranteed to all study participants that data would be used only for research.

### Conceptualization of Variables and Measurement

After reviewing the relevant literature and studies conducted by Sang et al. (2021) and Lin et al. (2020) in the socio-economic and CP perspective, the current study included living place (Suzhou, Wuhan); gender (male, female, others, and prefer not to answer); age (18–24, 25–34, 35–44, 45–54, 55–64, and 65 years or older); education (less than high school degree, high school degree, associate degree, bachelor's degree, and graduate degree); marital status (single-never married, married, or in a domestic partnership, widowed, divorced, and separated); and employment status (employed full time-including self-employed or homemaker, employed part-time-including self-employed or homemaker, unemployed, student, retired, and unable to work); and annual household income before taxes (Pre-COVID-19). The survey participants reported all the demographic variables used in the study. The CP affects everyday life, movements, trade, and business activities from local to global, which further impacts socio-economic lives of people (Haleem et al., 2020).

The CP and LP were used as independent variables in the current study. The CP was assessed by asking questions about risk perception and belief about the pandemic that emerged in Wuhan. The questions were based upon the belief about that how the COVID-19 emerged (Due to climate change, the CP created fear and anxiety, the belief that the COVID-19 is a threat to humanity, and the importance have health and well-being as a top priority in everyone's life after COVID-19). As a result, the lockdown was initially imposed to contain the transmission of the pandemic. Under the lockdown measure, several interventions were introduced among the general population for physical health well-being protection. In the present study, questions were asked about the LP interventions (stay at home, social distancing, wearing a facemask, wash hands with sanitizer, quarantine and avoid the areas where the pandemic is severe).

ER is the mediator variable according to the study objective. The questions related to ER in the current study were based upon the coping strategy aspects. The study participants were asked to report the strategies or steps (getting comfort and understanding from someone, use the substance to make myself feel better, accept the reality of fact and learned to live with it safely, maintaining positive thinking, to do physical exercise to release stress and anxiety and look for creative ways to alter the problematic situation) which they used during the lockdown period to regulate the emotions for psychological, physical, and overall health well-being. Infectious disease outbreaks are one of the most daunting conditions to deal with emotionally. Physical and emotional well-being of individuals is jeopardized as they must plan for an uncertain event. Since there is no definite time limit for the conclusion of infectious disease outbreaks, people feel at risk all the time (Bavel et al., 2020).

The dependent variables in this study were PH, PsH, PA, and OW during the CP lockdown. The question statements under the Likert scale for each dependent variable is based on the PH (during the COVID-19: Have you maintained personal hygiene for disease prevention? Have you been leading an active lifestyle during the COVID-19 lockdown? Did you have a healthy diet during the pandemic lockdown? During COVID-19, have you maintained physical fitness? and During COVID-19, did you have a normal sleep of 8 h?); PsH (During COVID-19 lockdown, have you experienced anxiety, bipolar disorder, insomnia, substance abuse or addiction, depression, and mental stress?); OW (During the CP, your PH remained stable, your PsH remained stable, your lifestyle remained active, your financial situation remained stable and your emotional health remained stable); and PA (use PA to cope with the health maintenance difficulties you faced, encourage others, including your family members, to do physical activities, PA levels during the lockdown period decrease, PA levels during the lockdown period increase and your PA levels during the lockdown period were almost the same). Thus, HRQoL is based on physical, psychological, and OW. Therefore, HRQoL is a multidimensional paradigm in public health and is based on various aspects, such as PH, PsH, and PA (Sitlinger and Zafar, 2018).

### Statistical Analysis

For analyzing the collected data for this study, the Smart-PLS 3.2.9 and SPSS 23 software were used (Ringle et al., 2015). The statistical analysis was based on two parts; univariate and multivariate. Under the univariate analysis, the demographic information of the survey participants was analyzed. And under the multivariate analysis, the structural equation model (SEM) technique was applied to examine the relationship between the study variables. Two-step techniques were used under the SEM for analyzing the data. The first step was the measurement model for checking construct validity, reliability, and convergent validity (CV). In the second step, the structural model was developed to test the hypothesis (Anderson and Gerbing, 1992; Hair et al., 2017). A robust, scalable, and advanced method for creating a significant statistical model is the Smart-PLS research design. The function of the Smart PLS-SEM helps achieve the intended objective (Abbas et al., 2019). The SEM in this study is based on six observed variables, as shown in Figure 1 (conceptual model), to assess the HRQoL. The CP and LP are the independent variables, while ER is considered a mediator variable. Furthermore, PH, PsH, PA, and OW are considered as dependent variables.

## Results

### Univariate Analysis

#### Demographic Characteristics of the Survey Participants (N = 2,200)

Table 1 shows the demographic characteristics of the survey participants. The age statistics show that majority of the survey participants (~37.73%) belonged to the age group of 18–24 years, ~18.82% belonged to the age group of 45–54 years, ~17.73% belonged to the age group of 35–44 years, ~10.77% belonged to the age group of +65 years, and ~8.77% belonged to the age group of 25–34 years, while only ~6.18% belonged to the age group of 55–64 years. In the context of gender distribution, the majority of the survey participants (~49.82%) were female, with a slight difference of ~49.36% being male, and only ~0.59% preferred not to answer about their gender identity. In comparison, only ~0.23% were others. Table statistics show that majority of the survey participants (~50.32%) were married or in a domestic partnership, ~41.73% were single (never married), ~4.91% were widowed, and ~2.64% were divorced. In comparison, only ~0.41% were separated. The educational background shows that the majority of the survey participants (27.04%) had a graduate degree, ~26.41% had a bachelor degree, ~22.32% had less than high school degree, ~14.68% had an associate degree, and ~9.54% had high school level education.

**Table 1:** Demographic characteristics of the survey participants.

**City wise demographics**	**Category**	**Suzhou (*N* = 1,034)**	**Wuhan (*N* = 1,166)**	**Overall (*N* = 2,200)**
Age (years)	18–24	316 (30.56%)	514 (44.08%)	830 (37.73%)
	25–34	172 (16.63%)	21 (1.80%)	193 (8.77%)
	35–44	253 (24.47%)	137 (11.75%)	390 (17.73%)
	45–54	199 (19.25%)	215 (18.44%)	414 (18.82%)
	55–64	77 (7.45%)	59 (5.06%)	136 (6.18%)
	65 or older	17 (1.64%)	220 (18.87%)	237 (10.77%)
Gender	Male	503 (48.65%)	583 (50.0%)	1,086 (49.36%)
	Female	524 (50.68%)	572 (49.05%)	1,096 (49.82%)
	Others	2 (0.19%)	3 (0.26%)	5 (0.23%)
	Prefer not to answer	5 (0.48%)	8 (0.69%)	13 (0.59%)
Marital status	Single (never married)	400 (38.68%)	518 (49.57%)	918 (41.73%)
	Married, or in a domestic partnership	595 (57.54%)	512 (43.91%)	1,107 (50.32%)
	Widowed	11 (1.06%)	97 (8.32%)	108 (4.91%)
	Divorced	22 (2.13%)	36 (3.09%)	58 (2.64%)
	Separated	6 (0.58%)	3 (0.26%)	9 (0.41%)
Education	Less than high school degree	137 (13.25%)	354 (30.36%)	491 (22.32%)
	High School	197 (19.05%)	13 (1.11%)	210 (9.54%)
	Associate degree	143 (13.83%)	180 (15.44%)	323 (14.68%)
	Bachelor's degree	167 (16.15%)	414 (35.51%)	581 (26.41%)
	Graduate degree	390 (37.72%)	205 (17.58%)	595 (27.04%)

### Multivariate Analysis

#### Assessment of the Measurement Model

**Figure 3:**
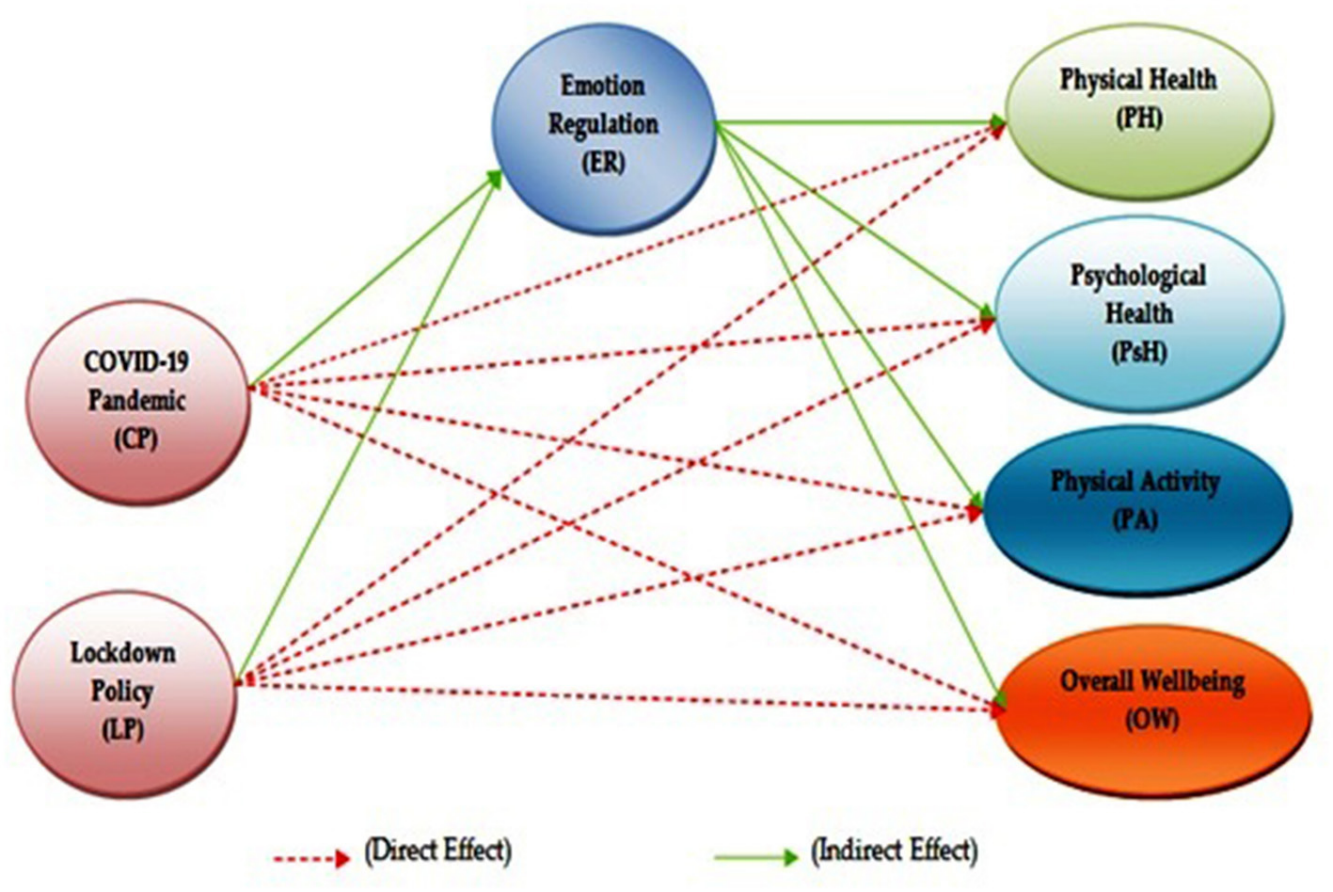
Factor loadings, path coefficient, and R-square result (PLS-algorithm).

We examined the internal consistency reliability, CV, and discriminant validity (see **Table 3**). Cronbach's alpha and composite reliability were utilized to evaluate the internal consistency of the measures used, which ranged from 0.823 to 0.966 and 0.876 to 0.975, respectively, thus surpassing the 0.70 cutoff in all the cases. For CV, the factor loadings of all the items and the average variance extracted (AVE) were conducted. CV was confirmed because loading of all the items was more than 0.6, and the AVE for all the constructs was more than 0.5 thresholds (Hair et al., 2017). All the mentioned results are presented in Table 2 and Figure 3.

**Table 2:** Construct validity and reliability (*N* = 2,200).

**Constructs**	**Items**	**FL**	**CA**	**CR**	**AVE**
COVID-19 pandemic	CP		0.966	0.975	0.907
	CP1	0.949			
	CP2	0.952			
	CP3	0.957			
	CP4	0.951			
Lockdown policy	LP		0.892	0.917	0.648
	LP1	0.744			
	LP2	0.767			
	LP3	0.808			
	LP4	0.839			
	LP5	0.843			
	LP6	0.825			
Emotion regulation	ER		0.880	0.909	0.625
	ER1	0.743			
	ER2	0.819			
	ER3	0.819			
	ER4	0.812			
	ER5	0.762			
	ER6	0.786			
Physical health	PH		0.823	0.876	0.587
	PH1	0.735			
	PH2	0.791			
	PH3	0.821			
	PH4	0.760			
	PH5	0.718			
Psychological health	PsH		0.959	0.967	0.830
	PsH1	0.878			
	PsH2	0.892			
	PsH3	0.936			
	PsH4	0.903			
	PsH5	0.925			
	PsH6	0.930			
Physical activity	PA		0.867	0.904	0.652
	PA1	0.789			
	PA2	0.829			
	PA3	0.759			
	PA4	0.851			
	PA5	0.807			
Overall well-being	OW		0.850	0.893	0.627
	OW1	0.776			
	OW2	0.835			
	OW3	0.831			
	OW4	0.811			
	OW5	0.698			

*FL, Factor Loadings; CA, Cronbach's Alpha; CR, Composite Reliability; AVE, Average Variance Extracted; CP, COVID-19 Pandemic; LP, Lockdown Policy; ER, Emotional Regulation; PH, Physical Health; PsH, Psychological Health; PA, Physical Activity; OW, Overall Well-being*.

#### Discriminant Validity

There are various approaches to determine discriminant validity, such as Fornell Lacker and Hetro Trait–Mono Trait (HTMT). Fornell Lacker is the first criterion that needs to confirm for discriminant validity. According to this process, the value of the square root of AVE of one construct must be higher than the value of inter-correlations between the constructs. This is because a construct must represent more variance with its items than others in the model. As depicted in Table 3, the square roots of the AVE of all constructs are more significant than their corresponding inter-correlation values. Henseler et al. (2016) proposed the HTMT method regarding discriminant validity, which confirms discriminant validity between each pair of variables. Table 4 shows that the HTMT values are below the threshold of 0.90.

**Table 3:** Discriminant validity: Fornell Larcker (*N* = 2,200).

**Constructs**	**CP**	**ER**	**LP**	**OW**	**PA**	**PH**	**PsH**
CP	**0.952**						
ER	0.057	**0.791**					
LP	0.032	−0.490	**0.805**				
OW	−0.046	0.668	−0.388	**0.792**			
PA	−0.014	0.727	−0.473	0.685	**0.808**		
PH	−0.138	0.591	−0.490	0.553	0.593	**0.766**	
PsH	0.567	−0.035	0.109	−0.138	−0.094	−0.122	**0.911**

*CP, COVID-19 Pandemic; ER, Emotion Regulation; LP, Lockdown Policy; OW; Overall well-being; PA, Physical Activity; PH, Physical Health; PsH, Psychological Health*.

**Table 4:** Discriminant validity (HTMT method) (*N* = 2,200).

**Items**	**CP**	**ER**	**LP**	**OW**	**PA**	**PH**	**PsH**
CP							
ER	0.070						
LP	0.071	0.537					
OW	0.060	0.771	0.435				
PA	0.039	0.825	0.527	0.803			
PH	0.150	0.693	0.555	0.656	0.701		
PsH	0.586	0.052	0.118	0.155	0.105	0.133	

*CP, COVID-19 Pandemic; Emotion Regulation; LP, Lockdown Policy; OW, Overall Wellbeing; PA, Physical Activity; PH, Physical Health; PsH, Psychological Health*.

#### Assessment of the Structural Equation Model

**Figure 4:**
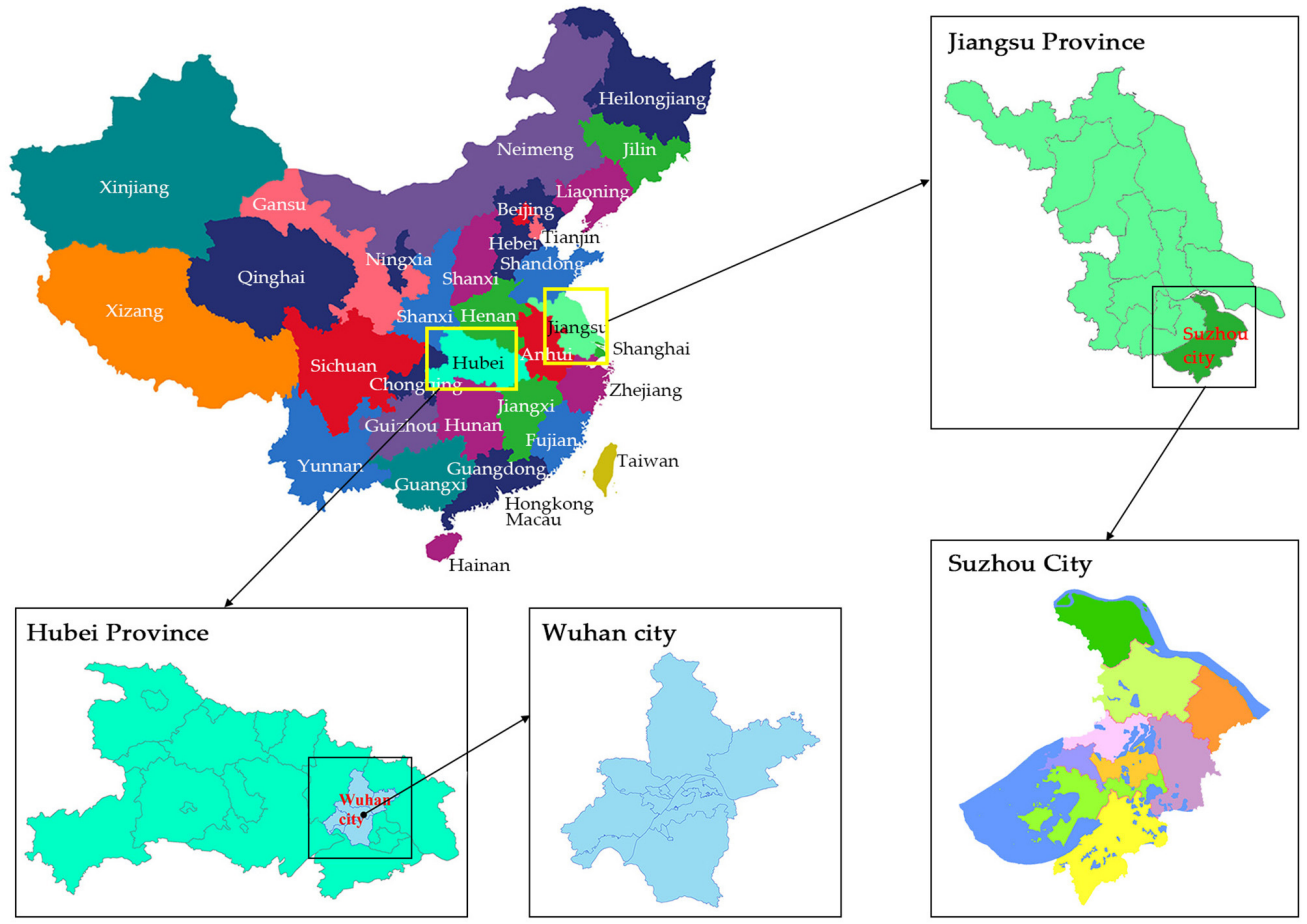
Bootstrapping results with inner model *t*-values.

According to Chin (2010), the structural model represents the theoretical model to evaluate the inner path model with structural equations. For the evaluation of the SEM in this research, the essential criteria used were path coefficient (β), coefficient of determination (R2) for an endogenous variable, effect size (f2), prediction relevance (q2), and multicollinearity (inner VIF) (Tenenhaus et al., 2005; Henseler et al., 2009; Götz et al., 2010). The threshold value and description for each benchmark are shown in Table 5 of the SEM. Table 5 presents the findings related to our direct hypotheses as well; in support of the first prediction, CP was significantly and negatively related to PH (β = −0.157, *t* = 9.444, *p* < 0.001, Table 6). Similarly, a significant relationship between second prediction CP and PsH was found (β = 0.779, *t* = 45.013, *p* < 0.001). For the third prediction (H3), the statistical analysis revealed that there is a significant negative relationship between CP and OW (β = −0.080, *t* = 5.261, *p* < 0.001). The fourth hypothesis (H4), the statistical analysis, revealed that there is a significant negative relationship between CP and PA (β = −0.047, *t* = 3.351, *p* < 0.001). Similarly, the other four paths, that is, LP and PH, LP and PsH, LP and OW, and LP and PA, were statistically significant with negative relationships as their *p*-values were <0.05. Therefore, the H1 to H8 was supported, which is presented in Table 6 and Figure 4.

**Table 5:** Assessment of structural equation model (*N* = 2,200).

***R*-square**	**Endogenous variables**	***R*** **-square**		***R*** **-square adjusted**			**Criteria**
	ER	0.245		0.245			0.26: Substantial, 0.13: Moderate, 0.02: Weak, Hair et al., 2017
	OW	0.458		0.457			
	PA	0.549		0.549			
	PH	0.426		0.426			
	PsH	0.330		0.329			
Effect size (*F*-square)	**Endogenous variables**	**CCR Q** ^2^ **(=1-SSE/SSO)**		**CCC Q** ^2^ **(=1-SSE/SSO)**			**Criteria**
	ER	0.150		0.471			0.26: Substantial, 0.13: Medium effect, 0.02: Small effect Hair et al., 2017
	OW	0.277		0.437			
	PA	0.347		0.472			
	PH	0.243		0.378			
	PsH	0.271		0.756			
Collinearity (Inner VIF)	**Exogenous variables**	**ER**	**OW**	**PA**	**PH**	**PsH**	**Criteria**
	CP	0.007	0.012	0.005	0.042	0.475	A value larger than (0) indicates Predictive Relevance Hair et al., 2017
	ER		0.563	0.722	0.299	0.001	
	LP	0.321	0.008	0.038	0.083	0.007	
Predictive relevance (*Q*-square)	**Exogenous variables**	**ER**	**OW**	**PA**	**PH**	**PsH**	**Criteria**
	CP	1.001	1.008	1.008	1.008	1.008	VIF ≤ 5.0, Hair et al., 2017
	ER		1.325	1.325	1.325	1.325	
	LP	1.001	1.323	1.323	1.323	1.323	

*ER, Emotion Regulation; OW, Overall Wellbeing; PA, Physical Activity; PH, Physical Health; PsH, Psychological Health; CP, COVID-19 Pandemic; LP, Lockdown Policy*.

**Table 6:** Path coefficient (direct effect) result (*N* = 2,200).

**Hypotheses**	**Original sample (O)**	**Sample mean (M)**	**SD**	***T***	***P*-values**	**Decision**
CP -> PH	−0.157	−0.158	0.017	9.444	0.001	Significant
CP -> PsH	−0.779	−0.566	0.013	45.013	0.001	Significant
CP -> OW	−0.080	−0.080	0.015	5.261	0.001	Significant
CP -> PA	−0.047	−0.046	0.014	3.351	0.001	Significant
LP -> PH	−0.251	−0.250	0.030	8.302	0.001	Significant
LP -> PsH	−0.076	−0.077	0.019	3.994	0.001	Significant
LP -> OW	−0.074	−0.072	0.026	2.816	0.005	Significant
LP -> PA	−0.150	−0.151	0.025	6.120	0.001	Significant

*CP, COVID-19 Pandemic; LP, Lockdown Policy; PH, Physical Health; PsH, Psychological Health; OW, Overall Wellbeing; PA, Physical Activity*.

Furthermore, the mediating effect of EC among the mediating path, such as CP -> ER -> PH, CP -> ER -> OW, CP -> ER -> PA, LP -> ER -> PH, LP -> ER -> OW, and LP -> ER -> PA were found statistically significant as the *t*-values were higher than 1.96 and the *p*-values were <0.05. Besides the confidence interval results for those relationships, LL and UL were negative values (i.e., “0” not in between), which also confirmed the mediation effect. Furthermore, all the mediation effects were found to be of partial mediation as their direct relationships were also significant. However, two mediation paths, such as CP -> ER -> PsH and LP -> ER -> PsH, revealed no significant mediation as their *p*-values were higher than 0.05 and zero “0” in between LL and UL. All the results are presented in Table 7 and Figure 4.

**Table 7:** Mediation (indirect effect) result (*N* = 2,200).

**Hypotheses**	**OS/Beta**	**LL**	**UL**	***T***	***P*-values**	**Decision**	**Mediation**
CP -> ER -> PH	−0.035	−0.054	−0.053	3.830	0.001	Significant	Partial
CP -> ER -> PsH	−0.002	−0.006	0.000	1.285	0.200	Not significant	No mediation
CP -> ER -> OW	−0.046	−0.026	−0.069	4.088	0.001	Significant	Partial
CP -> ER -> PA	−0.048	−0.026	−0.072	3.993	0.001	Significant	Partial
LP -> ER -> PH	−0.235	−0.279	−0.195	10.766	0.001	Significant	Partial
LP -> ER -> PsH	0.015	−0.002	0.034	1.631	0.104	Not significant	No mediation
LP -> ER -> OW	−0.313	−0.365	−0.265	12.368	0.001	Significant	Partial
LP -> ER -> PA	−0.323	−0.374	−0.274	12.639	0.001	Significant	Partial

*CP, COVID-19 Pandemic; LP, Lockdown Policy; ER, Emotion Regulation; PH, Physical Health; PsH, Psychological Health; OW, Overall Wellbeing; PA, Physical Activity; LL, Lower Limit; UL, Upper Limit*.

## Discussion

In the current study, we tried to explore the mediating role of ER. Furthermore, we assessed the impact of the CP and LP on PH–PsH, PA, and OW in the context of HRQoL. The Chinese government implemented strict measures for the safety of the Chinese people to contain the COVID-19 virus. Those measures have significant impacts on the HRQoL of the Chinese people.

The findings related to our first hypothesis confirmed our first prediction that CP was found significantly and negatively associated with PH. These findings are in line with the previous studies that reported the CP and the LPs negatively impacting the HRQoL during the COVID-19 epidemic in Morocco (Azizi et al., 2020). The CP affected PH by increasing inactive lifestyles, contributing to OW health-related problems (Krok and Zarzycka, 2020). The mediating effect of ER among the mediating paths was statistically significant as the *t*-values are higher than 1.96 and the *p*-values are <0.05. A study conducted in Mainland China reported that the effect of CP on emotional stability or the quality of life has negatively impacted China and many other parts of the world (Zhang and Ma, 2020). Our findings confirm that ER has a mediating effect because both lower and upper limits are negative values. The home confinement policy implementation regarding the containment of the COVID-19 negatively impacted the HRQoL (Lipskaya-Velikovsky, 2021). Similar results have been reported by Özdin and Bayrak Özdin (2020) and Rajkumar (2020) and found that ER, as a coping strategy, significantly impacts eliminating depression and anxiety. Cognitive ER as a coping strategy correlates with HRQoL (Dubey et al., 2020). ER strategies have a positive impact on psychological well-being (Extremera and Rey, 2014).

The closure of public spaces has negative impacts on the PA level. CP and LPs, directly and indirectly, impact socio-economic, psychological, and physical health well-being aspects of the human society (Sang et al., 2021). The statistical analysis of our study revealed a significant negative relationship between the CP and PA, which means PA level decreased among the targeted research population of our study. PA levels decreased from local to global levels across the world due to the CP lockdown. PA is a natural preventive measure against different non-communicable diseases and plays a vital role in maintaining the HRQoL (Dai and Menhas, 2020; Sánchez Castillo et al., 2020). The decreasing PA and inactive lifestyle were identified as vital issues during home confinement (Bentlage et al., 2020). PA levels decreased from local to global levels across the world due to the CP lockdown. Playgrounds, public parks, and recreational spaces closed due to implementing the COVID-19 LP to cutoff the transmission of the virus. In similar findings by López-Sánchez et al. (2020), it was reported that PA levels declined from about 60.6 to 48.9% among the Spanish population.

The PH, PsH, and OW of the Chinese people were affected due to the CP and lockdown measures. It is suggested that PA is the most suitable preventive measure against chronic anxiety. In the context of PsH, the COVID-19 outbreak increased the mental health issues of Wuhan residents reported by Bao et al. (2020), and a high rate of depression prevalence among young Chinese people has been found during the lockdown period (Huang and Zhao, 2020). General well-being, PH, and PsH are linked with PA. PA is also affected by the COVID-19 lockdown, negatively impacting the HRQoL. PA played a pivotal role in improving HRQoL, especially in the comorbidities reported by Hanke et al. (2020). In the context of PA related to our findings, similar results were reported in Greece and found adverse changes in PA due to the COVID-19 lockdown (Bourdas and Zacharakis, 2020). Our findings show that LP and PH, LP and PsH, LP and OW, and LP and PA also found statistically significant negative relationships as their *p*-values were <0.05. Similarly, a study conducted in Pakistan reported that the COVID-19 negatively influenced Pakistani students, further linked with high depression and mental anxiety (Salman et al., 2020). Likewise, a study conducted in Canada reported that outdoor and overall PA decreased due to the lockdown (Lesser and Nienhuis, 2020). In line with our findings, similar results have been reported by Narayanan et al. (2020) and found that the lifestyle of Indian people has been changed due to the COVID-19 lockdown measures, which further have negative impacts on HRQoL.

## Conclusions

COVID-19 was declared as a global pandemic by the WHO after many cases across China were confirmed. Due to the severity of the COVID-19, many countries worldwide introduced different measures to contain the pathogen of the COVID-19, such as lockdown of the whole country, smart lockdown, social distancing, and body temperature monitoring at home confinement. The preventive measures taken by different countries had an impact on the socio-economic perspectives to HRQoL (Azizi et al., 2020). Our results show that the CP and the LPs negatively impact the HRQoL among the Chinese population. Additionally, the mediating role of ER was found to significantly improve the HRQoL, such as CP -> ER -> PH, CP -> ER -> OW, CP -> ER -> PA, LP -> ER -> PH, LP -> ER -> OW, and LP -> ER -> PA. Stability and proper ER play a vital role in OW. PA is also important for OW, but due to the COVID-19 LPs across China, PA participation decreased. The mediating role of ER is critical for quality of life during the sudden emergence of an outbreak. ER can be defined as an individual's efforts to monitor and control their ecstatic response. It is suggested that ER intervention reduces the negative psychological impacts for improving quality of life. ER can function one's sentiments in their social environment effectively for quality of life.

## Study Limitations

The study has several limitations. The cross-sectional study design is a major limitation of this study. Furthermore, to participate in the survey, the respondent must be 18 years old and literate. The convenience sampling technique was used under the non-probability sampling method according to the objective and nature of the study. The study results cannot be generalized to the whole population because it is hard to replicate the convenience sample results.

## Data Availability Statement

The datasets presented in this article are not readily available because the data belongs to a multi-country ongoing project. Requests to access the datasets should be directed to the corresponding author RM.

## Ethics Statement

The studies involving human participants were reviewed and approved by the Ethics Committee of Soochow University (SUDA2020061H01) and the Ethics Committee of Wuhan Sports University (2020004). The study was conducted according to the Declaration of Helsinki's guidelines. The patients/participants provided their written informed consent to participate in this study.

## Author Contributions

JD is the principal investigator, while XS, YW, YC, and JH conducted an online survey and collected primary data. RM designed the study model, methodology, wrote the article, and did English editing. SK and MG guided psychological perspective. ZAS contributed to the discussion section with RM while MNA analyzed the data. BS, SM, and WI designed pictures, while XX did the proofreading and approved the manuscript. All authors contributed to the article and approved the submitted version.

## Conflict of Interest

The authors declare that the research was conducted in the absence of any commercial or financial relationships that could be construed as a potential conflict of interest.

## Publisher's Note

All claims expressed in this article are solely those of the authors and do not necessarily represent those of their affiliated organizations, or those of the publisher, the editors and the reviewers. Any product that may be evaluated in this article, or claim that may be made by its manufacturer, is not guaranteed or endorsed by the publisher.

## References

[B1] Abbas J. Mahmood S. Ali H. Ali R. M. Ali G. Aman J.. (2019). The effects of corporate social responsibility practices and environmental factors through a moderating role of social media marketing on sustainable performance of business firms. Sustainability 11:3434. 10.3390/su11123434

[B2] Al Zobbi M. Alsinglawi B. Mubin O. Alnajjar F. (2020). Measurement method for evaluating the lockdown policies during the COVID-19 pandemic. Int. J. Environ. Res. Public Health 17:5574. 10.3390/ijerph17155574 32748822 PMC7432619

[B3] Alwan N. A. Burgess R. A. Ashworth S. Beale R. Bhadelia N. Bogaert D.. (2020). Scientific consensus on the COVID-19 pandemic: we need to act now. Lancet. 396, e71–e72. 10.1016/S0140-6736(20)32153-X 33069277 PMC7557300

[B4] Anderson J. C. Gerbing D. W. (1992). Assumptions and comparative strengths of the two-step approach: comment on Fornell and Yi. Sociol. Methods Res. 20, 321–333. 10.1177/0049124192020003002

[B5] Anna F. L. (2020). Travel Ban Goes Into Effect in Chinese City of Wuhan as Authorities Try to Stop Coronavirus Spread. Available online at: https://www.washingtonpost.com/world/asia_pacific/nine-dead-as-chinese-coronavirus-spreads-despite-efforts-to-contain-it/2020/01/22/1eaade72-3c6d-11ea-afe2-090eb37b60b1_story.html (accessed January 1, 2020).

[B6] Azizi A. Achak D. Aboudi K. Saad E. Nejjari C. Nouira Y.. (2020). Health-related quality of life and behavior-related lifestyle changes due to the COVID-19 home confinement: dataset from a Moroccan sample. Data Brief 32:106239. 10.1016/j.dib.2020.106239 32868996 PMC7449885

[B7] Bao Y. Sun Y. Meng S. Shi J. Lu L. (2020). 2019-nCoV epidemic: address mental health care to empower society. Lancet 395, e37–e38. 10.1016/S0140-6736(20)30309-3 32043982 PMC7133594

[B8] Bavel J. Baicker K. Boggio P. S. Capraro V. Cichocka A. Cikara M.. (2020). Using social and behavioural science to support COVID-19 pandemic response. Nat. Hum. Behav. 4, 460–471. 10.1038/s41562-020-0884-z 32355299

[B9] Bentlage E. Ammar A. How D. Ahmed M. Trabelsi K. Chtourou H.. (2020). Practical recommendations for maintaining active lifestyle during the COVID-19 pandemic: a systematic literature review. Int. J. Environ. Res. Public Health 17:6265. 10.3390/ijerph17176265 32872154 PMC7503956

[B10] Bound A. F. (2018). This “modern epidemic:” loneliness as an emotion cluster and a neglected subject in the history of emotions. Emot. Rev. 10, 242–254. 10.1177/1754073918768876

[B11] Bourdas D. I. Zacharakis E. D. (2020). Impact of COVID-19 lockdown on physical activity in a sample of greek adults. Sports 8:139. 10.3390/sports8100139 33096721 PMC7589063

[B12] Brooks S. K. Webster R. K. Smith L. E. Woodland L. Wessely S. Greenberg N.. (2020). The psychological impact of quarantine and how to reduce it: rapid review of the evidence. Lancet 395, 912–920. 10.1016/S0140-6736(20)30460-8 32112714 PMC7158942

[B13] Chen P. Mao L. Nassis G. P. Harmer P. Ainsworth B. E. Li F. (2020). Coronavirus disease (COVID-19): the need to maintain regular physical activity while taking precautions. J. Sport Health Sci. 9, 103–104. 10.1016/j.jshs.2020.02.001 32099716 PMC7031771

[B14] Cheng S. K. W. Chong G. H. C. Chang S. S. Y. Wong C. W. Wong C. S. Y. Wong M. T. P.. (2006). Adjustment to severe acute respiratory syndrome (SARS): Roles of appraisal and post-traumatic growth. Psychol. Health. 21, 301–317. 10.1080/14768320500286450

[B15] Chin W. W. (2010). How to write up and report PLS analyses, in Handbook of Partial Least Squares, eds Esposito Vinzi V. Chin W. Henseler J. Wang H. (Berlin, Heidelberg: Springer), 655–690. 10.1007/978-3-540-32827-8_29

[B16] Dai J. Menhas R. (2020). Sustainable development goals, sports and physical activity: the localization of health-related sustainable development goals through sports in China: a narrative review. Risk Manag. Healthcare Pol. 13, 1419–1430. 10.2147/RMHP.S257844 32943959 PMC7478917

[B17] Duan L. Zhu G. (2020). Psychological interventions for people affected by the COVID-19 epidemic. Lancet Psychiatry 7, 300–302. 10.1016/S2215-0366(20)30073-0 32085840 PMC7128328

[B18] Dubey N. Podder P. Pandey D. (2020). Knowledge of COVID-19 and its influence on mindfulness, cognitive emotion regulation and psychological flexibility in the Indian Community. Front. Psychol. 11:589365. 10.3389/fpsyg.2020.589365 33281687 PMC7689361

[B19] Duy B. Yildiz M. A. (2014). Adaptation of the regulation of emotions questionnaire (REQ) for adolescents. Turkish Psychol. Counseling Guidance J. 5, 23–35. Available online at: https://www.semanticscholar.org/paper/Adaptation-of-the-Regulation-of-Emotions-(REQ)-for-Duy/c7c4161d2deedebb976b081ef3d2034ce319255b

[B20] Ekman P. E. Davidson R. J. (1994). The Nature of Emotion: Fundamental Questions. Oxford: Oxford University Press.

[B21] Extremera N. Rey L. (2014). Health-related quality of life and cognitive emotion regulation strategies in the unemployed: a cross-sectional survey. Health Qual. Life Outcomes 12:172. 10.1186/s12955-014-0172-6 25432102 PMC4263041

[B22] Götz O. Liehr-Gobbers K. Krafft M. (2010). Evaluation of structural equation models using the partial least squares (PLS) approach, in Handbook of Partial Least Squares, eds Esposito Vinzi V. Chin W. Henseler J. Wang H. (Berlin, Heidelberg: Springer), 691–711. 10.1007/978-3-540-32827-8_30

[B23] Goyal K. Chauhan P. Chhikara K. Gupta P. Singh M. P. (2020). Fear of COVID 2019: first suicidal case in India!. Asian J. Psychiatry 49:101989. 10.1016/j.ajp.2020.101989 32143142 PMC7130010

[B24] Gross J. J. (1998). The emerging field of emotion regulation: an integrative review. Rev. Gen. Psycho. 2, 271–299. 10.1037/1089-2680.2.3.271

[B25] Gross J. J. (2013). Emotion regulation: taking stock and moving forward. Emotion 13, 359–365. 10.1037/a0032135 23527510

[B26] Gross J. J. Munoz R. F. (1995). Emotion regulation and mental health. Clin. Psychol. Sci. Pract. 2, 151–164. 10.1111/j.1468-2850.1995.tb00036.x

[B27] Hair J. F. Hult G. T. M. Ringle C. M. Sarstedt M. Thiele K. O. (2017). Mirror, mirror on the wall: a comparative evaluation of composite-based structural equation modeling methods. J. Acad. Market. Sci. 45, 616–632. 10.1007/s11747-017-0517-x

[B28] Haleem A. Javaid M. Vaishya R. (2020). Effects of COVID−19 pandemic in daily life. Curr. Med. Res. Pract. 3:11. 10.1016/j.cmrp.2020.03.011 32292804 PMC7147210

[B29] Hanke A. A. Sundermeier T. Boeck H. T. Schieffer E. Boyen J. Braun A. C.. (2020). Influence of officially ordered restrictions during the first wave of COVID-19 pandemic on physical activity and quality of life in patients after kidney transplantation in a telemedicine based aftercare program—A KTx360° sub study. Int. J. Environ. Res. Public Health 17:9144. 10.3390/ijerph17239144 33297529 PMC7730551

[B30] Hawkley L. C. Capitanio J. P. (2015). Perceived social isolation, evolutionary fitness and health outcomes: a lifespan approach. Philos. Trans. Royal Soc. B Biol. Sci. 370:20140114. 10.1098/rstb.2014.0114 25870400 PMC4410380

[B31] Henseler J. Hubona G. Ray P. A. (2016). Using PLS path modeling in new technology research: updated guidelines. Indus. Manag. Data Syst. 2015:382. 10.1108/IMDS-09-2015-0382

[B32] Henseler J. Ringle C. M. Sinkovics R. R. (2009). The Use of Partial Least Squares Path Modeling in International Marketing. In New Challenges to International Marketing. Manchester: Emerald Group Publishing Limited. 10.1108/S1474-7979(2009)0000020014

[B33] Holmes E. A. O'Connor R. C. Perry V. H. Tracey I. Wessely S. Arseneault L.. (2020). Multidisciplinary research priorities for the COVID-19 pandemic: a call for action for mental health science. Lancet Psychiatry 7, 547–560. 10.1016/S2215-0366(20)30168-1 32304649 PMC7159850

[B34] Hossain M. M. Sultana A. Purohit N. (2020). Mental health outcomes of quarantine and isolation for infection prevention: a systematic umbrella review of the global evidence. SSRN 2020:3561265. 10.21203/rs.3.rs-25647/v1 32512661 PMC7644933

[B35] Huang Y. Zhao N. (2020). Generalized anxiety disorder, depressive symptoms and sleep quality during COVID-19 epidemic in China: a web-based cross-sectional survey. J. Med. Sci. 23, 15–19. 10.21203/rs.3.rs-17172/v1 32325383 PMC7152913

[B36] Julie B. (2020). Tips for Controlling Your Emotions During COVID-19. Available online at: https://firstthings.org/tips-for-controlling-your-emotions-during-covid-19/ (accessed January 20, 2021).

[B37] Krok D. Zarzycka B. (2020). Risk perception of COVID-19, meaning-based resources and psychological well-being amongst healthcare personnel: the mediating role of coping. J. Clin. Med. 9:3225. 10.3390/jcm9103225 33050068 PMC7599885

[B38] Leahy R. L. Tirch D. Napolitano L. A. (2011). Emotion Regulation in Psychotherapy: A Practitioner's Guide. Washington, DC: American Psychological Association.

[B39] Leppin A. Aro A. R. (2009). Risk perceptions related to SARS and avian influenza: theoretical foundations of current empirical research. Int. J. Behav. Med. 16, 7–29. 10.1007/s12529-008-9002-8 19214752 PMC7090865

[B40] Lesser I. A. Nienhuis C. P. (2020). The impact of COVID-19 on physical activity behavior and well-being of Canadians. Int. J. Environ. Res. Public Health 17:3899. 10.3390/ijerph17113899 32486380 PMC7312579

[B41] Li J. B. Yang A. Dou K. Wang L. X. Zhang M. C. Lin X. (2021). Chinese public's knowledge, perceived severity, and perceived controllability of the COVID-19 and their associations with emotional and behavioural reactions, social participation, and precautionary behaviour: a national survey. PsyArXiv. 10.31234/osf.io/5tmsh 33087109 PMC7576982

[B42] Li W. Yang Y. Liu Z. H. Zhao Y. J. Zhang Q. Zhang L.. (2020). Progression of mental health services during the COVID-19 outbreak in China. Int. J. Biol. Sci. 16, 1732–1738. 10.7150/ijbs.45120 32226291 PMC7098037

[B43] Lin Y. Zhong P. Chen T. (2020). Association between Socio-economic Factors and the COVID-19 outbreak in the 39 well-developed cities of China. Front. Public Health 8:546637. 10.3389/fpubh.2020.546637 33194948 PMC7662384

[B44] Lipskaya-Velikovsky L. (2021). COVID-19 isolation in healthy population in israel: challenges in daily life, mental health, resilience, and quality of life. Int. J. Environ. Res. Public Health 18:999. 10.3390/ijerph18030999 33498662 PMC7908389

[B45] López-Sánchez G. F. Pardhan S. Trott M. Sánchez-Castillo S. Jackson S. E. Tully M.. (2020). The association between physical activity and cataracts among 17,777 people aged 15–69 years residing in Spain. Ophthal. Epidemiol. 27, 272–277. 10.1080/09286586.2020.1730911 32070171

[B46] Low R. S. Overall N. Chang V. Henderson A. M. (2020). Emotion regulation and psychological and physical health during a nationwide COVID-19 lockdown. PsyArXiv. 10.31234/osf.io/pkncy 34843308

[B47] Muzi S. Sans,ò A. Pace C. S. (2021). What's happened to Italian adolescents during the COVID-19 pandemic? A preliminary study on symptoms, problematic social media usage, and attachment: relationships and differences with pre-pandemic peers. Front. Psychiatry 12:590543. 10.3389/fpsyt.2021.590543 33986698 PMC8110826

[B48] Narayanan L. Pandit M. Basu S. Karmakar A. Bidhan V. Kumar H.. (2020). Impact of lockdown due to COVID-19 outbreak: lifestyle changes and public health concerns in India. Preprints 2020060129. 10.20944/preprints202006.0129.v1

[B49] Ozamiz-Etxebarria N. Mondragon N. I. Santamaría M. D. Gorrotxategi M. P. (2020). Psychological symptoms during the two stages of lockdown in response to the COVID-19 outbreak: an investigation in a sample of citizens in Northern Spain. Front. Psychol. 11:1491. 10.3389/fpsyg.2020.01491 32625157 PMC7314923

[B50] Özdin S. Bayrak Özdin S. (2020). Levels and predictors of anxiety, depression and health anxiety during COVID-19 pandemic in Turkish society: the importance of gender. Int. J. Soc. Psychiatry 66, 504–511. 10.1177/0020764020927051 32380879 PMC7405629

[B51] Plümper T. Neumayer E. (2020). Lockdown policies and the dynamics of the first wave of the Sars-CoV-2 pandemic in Europe. J. Eur. Public Pol. 27, 1–21. 10.1080/13501763.2020.1847170

[B52] Qi M. Li P. Moyle W. Weeks B. Jones C. (2020). Physical activity, health-related quality of life, and stress among the Chinese adult population during the COVID-19 pandemic. Int. J. Environ. Res. Public Health 17:6494. 10.3390/ijerph17186494 32906604 PMC7558071

[B53] Rajkumar R. P. (2020). COVID-19 and mental health: a review of the existing literature. Asian J. Psychiatry 52:102066. 10.1016/j.ajp.2020.102066 32302935 PMC7151415

[B54] Ringle C. M. Wende S. Becker J. M. (2015). SmartPLS 3. Boenningstedt: SmartPLS; GmbH.

[B55] Rogowska A. M. Pavlova I. Kuśnierz C. Ochnik D. Bodnar I. Petrytsa P. (2020). Does physical activity matter for the mental health of university students during the COVID-19 pandemic? J. Clin. Med. 9:3494. 10.3390/jcm9113494 33138047 PMC7693909

[B56] Rubin G. Potts H. Michie S. (2010). The impact of communications about swine flu (influenza A H1N1v) on public responses to the outbreak: results from 36 national telephone surveys in the UK. Health Technol. Assess 14, 183–266. 10.3310/hta14340-03 20630124

[B57] Saarni C. (2008). The interface of emotional development with social context, in Handbook of emotions, eds Lewis M. Haviland-Jones J. M. Barrett L. F. (The Guilford Press), 332–347. Available online at: https://psycnet.apa.org/record/2008-07784-020

[B58] Salman M. Asif N. Mustafa Z. U. Khan T. M. Shehzadi N. Tahir H.. (2020). Psychological impairment and coping strategies during the COVID-19 pandemic among students in Pakistan: a cross-sectional analysis. Disast. Med. Public Health Preparedness 1, 1–22. 10.1017/dmp.2020.397 33087206 PMC7873451

[B59] Sánchez Castillo S. Smith L. Díaz Suárez A. López Sánchez G. F. (2020). Analysis of physical activity and comorbidities in spanish asthmatics. Sustainability 12:5256. 10.3390/su12135256

[B60] Sander D. Armony J. Vuilleumier P. (2013). The Cambridge Handbook of Human affective Neuroscience. Cambridge University Press. 5–3.

[B61] Sang X. Menhas R. Saqib Z. A. Mahmood S. Weng Y. Khurshid S.. (2021). The psychological impacts of COVID-19 home confinement and physical activity: a structural equation model analysis. Front. Psychol. 11:614770. 10.3389/fpsyg.2020.614770 33519638 PMC7843378

[B62] Saqib Z. A. Dai J. Rashid M. Shahid M. Karim M. Sang X.. (2020). Physical activity is a medicine for non-communicable diseases: a survey study regarding the perception of physical activity impact on health wellbeing. Risk Manag. Healthcare Pol. 13:2949. 10.2147/RMHP.S280339 33335436 PMC7737939

[B63] Shi L. Lu Z. A. Que J. Y. (2020). Prevalence of and risk factors associated with mental health symptoms among the general population in China during the coronavirus disease 2019 pandemic. J. Am. Med. Assoc. Netw. Open 3:14053. 10.1001/jamanetworkopen.2020.14053 32609353 PMC7330717

[B64] Sitlinger A. Zafar S. Y. (2018). Health-related quality of life: the impact on morbidity and mortality. Surg. Oncol. Clin. North America 27, 675–684. 10.1016/j.soc.2018.05.008 30213412 PMC6428416

[B65] Sloan E. Hall K. Moulding R. Bryce S. Mildred H. Staiger P. K. (2017). Emotion regulation as a transdiagnostic treatment construct across anxiety, depression, substance, eating and borderline personality disorders: a systematic review. Clin. Psychol. Rev. 57, 141–163. 10.1016/j.cpr.2017.09.002 28941927

[B66] Tenenhaus M. Vinzi V. E. Chatelin Y. M. Lauro C. (2005). PLS path modeling. Computat. Statist. Data Anal. 48, 159–205. 10.1016/j.csda.2004.03.005

[B67] Tsamakis K. Triantafyllis A. S. Tsiptsios D. Spartalis E. Mueller C. Tsamakis C.. (2020). COVID-19 related stress exacerbates common physical and mental pathologies and affects treatment. Exp. Therapeut. Med. 20, 159–162. 10.3892/etm.2020.8671 32509006 PMC7271730

[B68] Velotti P. Civilla C. Rogier G. Beomonte Zobel S. (2021a). A fear of COVID-19 and PTSD symptoms in pathological personality: the mediating effect of dissociation and emotion dysregulation. Front. Psychiatry 12:590021. 10.3389/fpsyt.2021.590021 33833698 PMC8021772

[B69] Velotti P. Rogier G. Beomonte Zobel S. Castellano R. Tambelli R. (2021b). Loneliness, emotion dysregulation, and internalizing symptoms during coronavirus disease 2019: a structural equation modeling approach. Front. Psychiatry 11:581494. 10.3389/fpsyt.2020.581494 33488417 PMC7819957

[B70] Wang C. Pan R. Wan X. Tan Y. Xu L. Ho C. S.. (2020). Immediate psychological responses and associated factors during the initial stage of the 2019 coronavirus disease (COVID-19) epidemic among the general population in China. Int. J. Environ. Res. Public Health 17:1729. 10.3390/ijerph17051729 32155789 PMC7084952

[B71] Yang Z. J. (2016). Altruism during Ebola: risk perception, issue salience, cultural cognition, and information processing. Risk Anal. 36, 1079–1089. 10.1111/risa.12526 26660724

[B72] Zhang Y. Ma Z. F. (2020). Impact of the COVID-19 pandemic on mental health and quality of life among local residents in Liaoning Province, China: a cross-sectional study. Int. J. Environ. Res. Public Health 17:2381. 10.3390/ijerph17072381 32244498 PMC7177660

